# A matched pair analysis of outcomes after stapler-assisted pharyngeal closure following laryngectomy

**DOI:** 10.1017/S0022215124001269

**Published:** 2025-01

**Authors:** Antony Abraham Paulose, Rajiv Charles Michael, Natarajan Ramalingam, Jeyashanth Riju, Mahasampath Gowri S, Lisa Abraham, Jino Johns L, Manu Mathew, Meera Thomas, Aparna Irodi

**Affiliations:** 1 Department of Head and Neck Surgery, Christian Medical College, Vellore, India; 2 Department of Biostatistics, Christian Medical College, Vellore, India; 3 Department of Radiation Oncology, Christian Medical College, Vellore, India; 4 Department of Pathology, Christian Medical College, Vellore, India; 5 Department of Radiodiagnosis, Christian Medical College, Vellore, India

**Keywords:** laryngectomy, laryngeal cancer, surgical staplers, squamous cell carcinoma of head and neck, treatment outcome

## Abstract

**Objective:**

To compare perioperative and oncological outcomes between stapler and manual closure in patients undergoing total laryngectomy for advanced endolaryngeal squamous cell carcinoma.

**Methods:**

Patients with advanced endolaryngeal tumours operated between July 2017 and July 2023 were retrospectively dichotomised into stapler closure and manual closure cohorts and compared.

**Results:**

Seventy-one patients with a median age of 57 years were included in our study. The median surgical duration was 270 minutes for the manual closure cohort and 245 minutes for the stapler closure cohort. The pharyngo-cutaneous salivary fistula rate was 6 per cent less in the stapler closure cohort. The estimated mean survival was not significantly different 54.5 months (95 per cent, confidence interval 46.3–62.71) in the manual closure cohort versus 28.12 months (95 per cent, confidence interval 23.6–32.63) in the stapler closure cohort (*p* = 0.79).

**Conclusion:**

Stapler closure can be used in endolaryngeal tumours, and it reduces operating time, thus facilitating efficient utilisation of operation time with non-inferior oncological outcomes as compared to traditional manual closure.

## Introduction

Surgical treatment in the form of total laryngectomy is the treatment of choice for advanced laryngeal malignancies and in failed organ-preservation strategies.^1^ Common complications following laryngectomy include pharyngo-cutaneous salivary fistula, surgical site infections, cricopharyngeal spasm and atony, and stoma-associated complications including stomal stenosis and dehiscence. Pharyngo-cutaneous salivary fistula, the most common complication, not only depends on the pharyngeal closure technique but is also influenced by the mucosal viability, prior chemoradiation history and nutritional status of the patient.^2–4^

Recent literature has shown a gradual decrease in the incidences of pharyngo-cutaneous salivary fistula from as high as 60–70 per cent to 5–10 per cent.^5^ Our prior data suggest an overall incidence of pharyngo-cutaneous salivary fistula in 16 per cent of cases undergoing total laryngectomy and manual closure pharyngoplasty.^6^ Stapler-assisted closure, in both closed and semi-closed techniques, achieves a water-tight closure without contamination of pharyngeal secretion, with minimal trauma to remnant mucosa, thus reducing the incidence of pharyngo-cutaneous salivary fistula and surgical site infections.^7–9^ Stapler-assisted closure requires a prior margin assessment to establish a purely endolaryngeal extent since margin adequacy is crucial while engaging the stapler device.^5,10^

Multiple studies in the literature have looked at functional aspects of stapler-assisted closure but bereft of an oncological safety profile.^7,11–14^ This study was designed to look at both the functional and oncological safety profiles of stapler-assisted pharyngeal closure compared with traditional manual closure as a matched-pair analysis.

## Materials and methods

**Figure 1.**
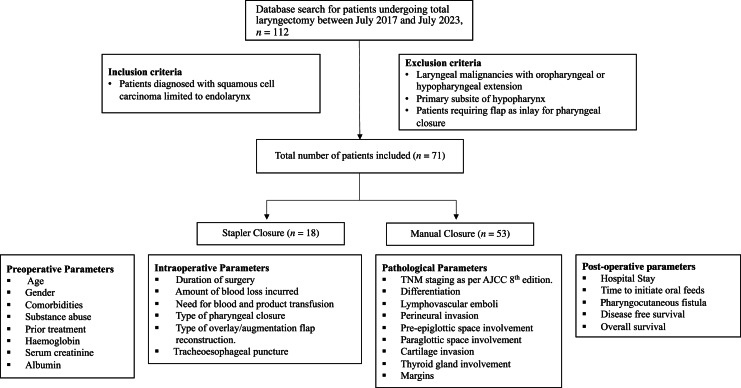
Study flow chart. TNM = tumour, node, metastasis; AJCC = American Joint Committee on Cancer.

This retrospective matched-pair cohort study was conducted with prior institutional review board clearance (IRB Minute No 15485 (RETRO) dated 28 June 2023) and recruited all eligible patients with diagnosed advanced endo-laryngeal squamous cell carcinoma without pharyngeal mucosal extension between July 2017 and July 2023. The study included patients who underwent total laryngectomy in a primary or salvage setting in the Department of Head and Neck Surgery (Figure 1). All patients underwent clinico-radiological evaluation, multi-disciplinary team discussion and pre-operative endoscopic disease assessment as a routine. Patients were staged as per the American Joint Committee on Cancer (AJCC) 8th edition. An on-table endoscopic reassessment or direct laryngoscopy was performed under general anaesthesia prior to laryngectomy for reaffirming the disease extent. Following total laryngectomy, traditional repair of the neopharynx was done using continuous Connell suture with 3-0 absorbable polyglactin suture material or 3-0 braided polyester suture material. Since March 2020, closed stapler-closure technique was used when feasible and manual suture technique was used when oncological clearance was in doubt. Tracheoesophageal puncture with voice prosthesis insertion was done as per the decision of the patient following voice rehabilitation counselling. Cricopharyngeal myotomy was done in all patients. Linear stapler 60 mm TX60 with 4 mm by 4.8 mm reload (®Ethicon Endosurgery, Johnson & Johnson, New Brunswick, NJ) was used in cases undergoing stapler closure.

Electronic medical reports of outpatient visits and inpatient records were scrutinised and those with endolaryngeal tumours were recruited to the study. Collected data parameters included patient general characteristics, laboratory investigations, surgery details, histopathological findings, adjuvant therapy details, post-operative details and follow up of patient.

Operation time was defined as the duration between incision time and wound closure. Pharyngo-cutaneous salivary fistula was suspected in the presence of progressive neck oedema or neck wound dehiscence with associated mucoid or mucopurulent discharge.

All cases were discussed by the Multi-Disciplinary Tumour Board at each treatment-decision phase starting pre-operatively, prior to adjuvant therapy and at diagnosis of a suspected disease failure. Overall survival was defined as the period between the date of biopsy to the last follow up.

The patients were dichotomised based on the type of closure into two cohorts: stapler-assisted closure and manual closure. Both cohorts were matched in a 3:1 ratio for age, co-morbidities, salvage surgery and tumour stage, as these variables predominantly influence the outcomes in question. Data were collected on SPSS (IBM SPSS Version 20). Continuous variables were compared with an independent sample *t*-test, and categorical variables were compared by a non-parametric independent sample median test. Statistical analysis was done in SPSS and R (Version 4.3.1). A two-sided *p*-value of less than 0.05 was considered significant.

## Results

Seventy-one patients were included in our study, with 53 patients undergoing manual suturing for pharyngeal closure and 18 patients undergoing stapler-assisted closure. The median age of the study population was 57 years. The mean age of patients in the stapler group was 58 ± 7 years, and the mean age of patients in the manual closure group was 57 ± 9 years (*p* = 0.98; Table 1). There was only one female patient in the study population who underwent manual closure. Most patients were in an advanced stage in the stapler group (III = 39 per cent, IVA = 39 per cent, IVB = 11 per cent) and in the manual closure group (III = 31 per cent, IVA = 50 per cent, IVB = 6 per cent).

**Table 1.** Comparison of demographic details and tumour characteristics between stapler closure and manual closure groups

Parameter	Stapler Closure Group (N = 18)	Manual Closure Group (N = 53)	*p*-value
Age, mean in years (SD)	58 (7)	57 (9)	
<55 years	6 (33%)	23 (43%)	0.45
≥55 years	12 (67%)	30 (57%)	
Gender			
Male	18 (100%)	52 (98%)	0.56
Female	0 (0%)	1 (2%)	
Comorbidities			
No	12 (67%)	23 (43%)	0.088
Yes	6 (33%)	30 (57%)	
Prior Irradiation			
No	14 (78%)	29 (55%)	0.084
Yes	4 (22%)	24 (45%)	
Substance Use History			
Former Smoker	10 (56%)	40 (75%)	0.19
Former Alcohol user	4 (22%)	13 (25%)	0.84
Smokeless Tobacco	6 (33%)	11 (21%)	0.28
Mean Haemoglobin in g/dL (SD)	11.54 (1.55)	11.33 (1.37)	0.60
Mean serum Albumin in g/dL	3.79 (0.50)	3.87 (0.62)	0.68
Mean serum Creatinine in mg/dL	0.95 (0.23)	0.86 (0.17)	0.072
T-stage			0.94
T0	0 (0%)	1 (2%)	
T2	2(11%)	6 (12%)	
T3	8 (44%)	21 (40%)	
T4a	8 (44%)	24 (46%)	
N-stage			0.67
N0	14 (78%)	35 (71%)	
N1	0 (0%)	1 (2%)	
N2a	0 (0%)	2 (4%)	
N2b	0 (0%)	4 (8%)	
N2c	2 (11%)	4 (8%)	
N3b	2 (11%)	3 (6%)	
Stage			0.83
0	0 (0%)	1 (2%)	
II	2 (11%)	6 (12%)	
III	7 (39%)	16 (31%)	
IVA	7 (39%)	26 (50%)	
IVB	2 (11%)	3 (6%)	
Grade of Differentiation			0.49
WDSCC	1 (6%)	6 (11.3%)	
MDSCC	15 (83%)	43 (81.1%)	
PDSCC	2 (12%)	4 (7.6%)	
Lymphovascular Emboli			
No	15 (83%)	44 (86%)	0.76
Yes	3 (17%)	7 (14%)	
Perineural Invasion			
No	13 (72%)	34 (67%)	0.66
Yes	5 (28%)	17 (33%)	
Paraglottic Space Involvement			
No	3 (17%)	12 (24%)	0.54
Yes	15 (83%)	39 (76%)	
Pre-epiglottic Space Involvement			
No	18 (100%)	44 (86%)	0.097
Yes	0 (0%)	7 (14%)	
Thyroid Cartilage Involvement			
No	6 (33%)	29 (56%)	0.10
Yes	12 (67%)	23 (44%)	
Cricoid Cartilage Involvement			
No	14 (78%)	36 (69%)	0.49
Yes	4 (22%)	16 (31%)	
Thyroid Gland Involvement			
No	17 (100%)	34 (83%)	0.069
Yes	0 (0%)	7 (17%)	

SD = standard deviation; WDSCC = well-differentiated squamous cell carcinoma; MDSCC = moderately differentiated squamous cell carcinoma; PDSCC = poorly differentiated squamous cell carcinoma

Both groups were matched in terms of age (*p* = 0.45), gender (*p* = 0.56), comorbidities (*p* = 0.089), prior history of substance abuse and irradiation (*p* = 0.08), and laboratory and histopathological parameters. The majority of the patients (57 per cent *vs* 23 per cent) who underwent manual closure had a prior history of radiotherapy, although it did not meet statistical significance. In the stapler closure group, it was noted that 67 per cent had thyroid cartilage involvement and 22 per cent had cricoid cartilage invasion, but none of the patients had pre-epiglottic space invasion.

There were no stapler device-related technical failures in any patients who underwent stapler-assisted pharyngeal closure. None of the patients underwent change in the pharyngeal closure method following intra-operative assessment. Median operation duration was 270 minutes (interquartile range: 225–310 min) in manual suturing group compared to 245 minutes (interquartile range: 220–320 min) in stapler-assisted closure (*p* = 0.50). The surgery cost incurred in stapler closure was 1.002 times that in manual closure. No cases of early-onset pharyngeal leaks were noted in either group within the first five days from surgery. The pharyngo-cutaneous salivary fistula rates were slightly higher in the manual suturing group as compared to stapler closure, although this difference was not statistically significance (17 per cent *vs* 11 per cent, respectively; *p* = 0.55). There was no difference between the two cohorts in terms of duration of hospital stay, time taken for initiation of oral feeds, time taken for initiation of adjuvant therapy, operating time, cost of surgery, or blood loss (Table 2). Primary tracheoesophageal puncture with voice prosthesis insertion was done in three patients in the stapler group and none had tracheoesophageal puncture failures. One patient in the manual closure group required multiple sitting of oesophageal dilatation for swallowing difficulty. None of the patients in the stapler group had swallowing difficulty.

**Table 2.** Comparison of Surgery parameters and functional outcomes between stapler closure and manual closure groups

Parameter	Stapler Closure Group (N = 18)	Manual Closure Group (N = 53)	*p*-value
Operating time, minutes (IQR)	245 (220–320)	270 (225–310)	0.5
Blood loss, mL (IQR)	300 (250–700)	400 (275–500)	0.89
Blood Transfusion			
No	17 (94%)	48 (91%)	0.80
Yes	1 (6%)	5 (9%)	
Flap Reconstruction			
None	12 (67%)	36 (68%)	0.18
Yes (as overlay)	6 (33%)	17 (32%)	
TEP-VP insertion			
No	15 (83%)	13 (25%)	<0.001
Yes	3 (17%)*	40 (75%)	
TEP Failure			
No	3 (100%)	36 (90%)	0.57
Yes	0 (0%)	4 (10%)	
Hospital Stay days (min, max)	5 (4,7)	5 (4,6)	0.27
Time to initiate oral feeds days (min, max)	13 (8,18)	13 (11,15)	0.97
PCF			
No	16 (89%)	44 (83%)	0.55
Yes	2 (11%)	9 (17%)	

IQR = interquartile range; TEP = tracheoesophageal puncture; TEP-VP = tracheoesophageal puncture with voice prosthesis; * = primary TEP; PCF = pharyngo-cutaneous salivary fistula

All patients in the stapler closure group had a clear margin on histopathological examination (n = 18; 100 per cent). Three (6 per cent) patients had a positive margin in the manual closure group. Sixteen patients (30.2 per cent) in the manual closure group underwent overlay flap reconstruction, the majority of which were sternocleidomastoid muscle overlay flaps (*n* = 11; 21 per cent), followed by four patients with pectoralis major pedicled flaps, and supraclavicular artery island flap in one patient. Six patients (33.3 per cent) in the stapler closure group underwent overlay flaps, of these, three patients (17 per cent) underwent supraclavicular artery island flap overlays, two underwent sternocleidomastoid muscle flaps, and one underwent a pectoralis major pedicled flap overlay. Twelve (67 per cent) of the patients were closed without a flap cover.

With a median follow up of 22.28 months in the study population, 19.7 per cent of the patients developed disease recurrence. The estimated mean overall survival was 28.12 months (95 per cent; CI 23.6–32.63) for the stapler-assisted closure cohort and 54.5 months (95 per cent; CI 46.3–62.71) for the manual closure cohort (*p* = 0.79), with comparable oncological outcomes and tumour control rates (Table 3).

**Table 3.** Comparison of oncological outcomes between stapler closure and manual closure groups

Parameter	Stapler Closure Group (N = 18)	Manual Closure Group (N = 53)	*p*-value
Margin			
Negative	18 (100%)	50 (94%)	0.30
Positive	0 (0%)	3 (6%)	
Mean Lateral Margin			
Ipsilateral	13.11 ± 8.60 mm	10.0 ± 7.87 mm	0.16
Contralateral	18.61 ± 7.52 mm	16.64 ± 9.94 mm	0.45
Disease Status			
Disease free	15 (83%)	34 (64.5%)	0.56
Local Recurrence	0 (0%)	3 (5.7%)	
Regional Recurrence	1 (6%)	5 (9.4%)	
Distant Metastasis	2 (11%)	2 (3.6%)	
Second Primary	0 (0%)	1 (1.8%)	
Status Unknown	0 (0%)	8 (15%)	
Survival Status			
Alive	14 (82%)	31 (69%)	0.29
Dead	3 (18%)	14 (31%)	
Death unrelated to disease	2 (66.7%)	3 (21%)	
Mean Follow up			
Overall Survival months	51 (95%) (CI = 43.25–58.84)	29 (95%) (CI = 24.16–33.18)	

**Figure 2.**
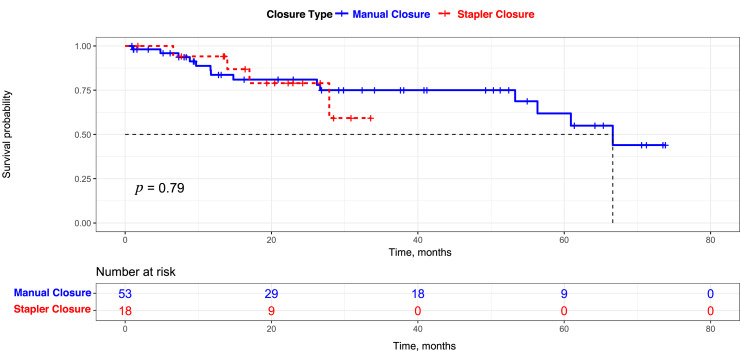
Kaplan–Meier plots comparing stapler closure and manual closure groups.

Eighty-three per cent (*n* = 15) of the stapler closure patients and 76 per cent (*n* = 34) of the patients in the manual closure group were disease free. One patient had a regional nodal recurrence, and two patients had distant metastasis to lung in the stapler group compared to three (7 per cent) local recurrences, five (11 per cent) regional recurrences and two (4 per cent) distant metastases in the manual closure group (Figure 2).

## Discussion

Mechanical stapling was initially used in abdominal surgeries. The earliest mention of the use of a stapler for laryngeal defect closure was in 1971.^15^ Mechanical stapling of pharyngeal defect can be done either by a closed or semi-closed technique. In the closed technique, the tracheal cut is made, and the laryngectomy specimen is completely skeletonised, separating it from the posterior pharyngeal/oesophageal wall, keeping only the mucosa intact. Vallecula mucosa is thinned, and the epiglottis is lifted up with the help of a Babcock forceps or folded inside using a cricoid hook passed through the tracheal stump, before the stapler is engaged. While the closed method is technically easier, it is rather a blind procedure in terms of margin assessment, and care should be taken so that the nasogastric tube, if placed, or epiglottis should not come in the engagement line of the linear stapler.^9^

The semi-closed technique gives the advantage of real-time surgical-margin assessment with the help of an endoscope and preventing entrapment of the epiglottis in the engagement line of the stapler. In this method, before engaging the stapler device, a small pocket is created in the mucosa of the vallecula, ideally in the midline, and an endoscope is introduced to assess the margins. The tip of the epiglottis is everted, and the stapler device can be engaged, achieving safe margins.^7^ We prefer doing a closed technique, having a safety margin assessment done using a direct laryngoscopy examination before the start of the procedure. This avoids mucosal breach and helps attain all the benefits of using a stapler closure. Thus, endolaryngeal tumours with or without minimal thyroid cartilage invasion are ideal candidates for stapler-assisted laryngectomy.^16^

Stapler closure techniques have been shown to improve pharyngo-cutaneous salivary fistula rates, decrease hospital stay, and achieve earlier initiation of oral feeds.^17–19^ Aires *et al*., in a systematic review of four studies, noted the incidence of pharyngo-cutaneous salivary fistula among those undergoing stapler closure to be 8.7 per cent compared to 22.9 per cent in those undergoing manual closure.^18^ A similar incidence rate (stapler closure 9.5 per cent *vs* manual closure 23.4 per cent) was noted for stapler closure in a recent meta-analysis by Chiesa-Estomba *et al*.^17^ Similar results were found in our study where pharyngo-cutaneous salivary fistula was noted in nine (16.98 per cent) of the 53 patients who underwent manual suturing and two (11.1 per cent) among the 18 patients with stapler closure. Various techniques have been adopted to decrease the incidence of pharyngo-cutaneous salivary fistula, such as tension-free manual closure techniques (namely T-shaped closure) or vertical closure and overlaying of a pedicled or a free flap.

In our study population, an overlay flap was used to secure the pharyngoplasty closure line as an added protection in 32.4 per cent of patients. The sternocleidomastoid muscle myogenous augmentation of the pharyngoplasty is advantageous in terms of shorter operation time as it belongs to the same field of surgery and is less bulky, thus avoiding a tractional force on the suture line. However, this flap closure may be unreliable in salvage settings and on the side where neck dissection is performed. Sternocleidomastoid muscle flap was used for augmentation in 11 of the manual closure cases, of which one case had a pharyngo-cutaneous salivary fistula, and in two patients of stapler closure. We also prefer de-epithelialised supraclavicular artery island flap as an overlay flap because it is less bulky and reliable. Pectoralis major pedicled myogenous flap overlay is used in patients undergoing salvage laryngectomy with significant post-radiotherapy-related changes in the neck.

The stapling techniques attempt to eliminate the surgeon factor in pharyngeal closure and achieve a water-tight closure line. In addition, stapler closure prevents undue manipulation of neopharyngeal mucosa, thereby reducing mucosal trauma and vascular insufficiency, and prevents contamination of the surgical site with pharyngeal secretions.

Moreover, stapler closure decreases surgery duration. The mean operating time saved in stapler closure was noted to be 40.67 minutes in a randomised control trial of 60 patients.^11^ Similarly, there was an 80-minute operation duration advantage noted in the systematic review by Aires *et al*.^18^ We noted the median operating time was 25 minutes less in the stapler closure group compared to the manual closure group, but it did not meet statistical significance (*p* = 0.5). Since stapler closure is a one-step shorter procedure, in practice, the total procedure time is more meaningful than assessing only the time for pharyngeal closure.

On considering the costs incurred, we have noted that the overall surgical costs were comparable. This could be due to the increased time taken for surgery, anaesthesia, and procedural charges in the manual closure group. In a prospective randomised control study by Ahmed *et al*., the costs incurred for stapler closure were 1.78 times that of the manual closure technique.^11^ The same was not noticed in our study. This might be because the cost incurred by the device might be overcome by reduced surgical duration related and anaesthesia costs.

Oncological safety is of paramount importance in malignancy resections. Most of the prior studies have not studied oncological margin safety and survival analysis for those undergoing stapler closure. Galli *et al*. noted 4.3 per cent of margins to be involved in their retrospective cohort of 46 patients undergoing stapler-assisted closure compared to 17.6 per cent involved margin rate in those undergoing manual closure.^12^ Similarly, Babu *et al*., in a retrospective study of 30 patients, noted 6.7 per cent of the patients with involved margins.^20^

The present study noted three (5.7 per cent) patients to have a positive margin in the manual closure group and none in the stapler closure group (*p* = 0.3). Mean lateral margins achieved were 13.11 ± 8.6 mm in the stapler closure group versus 10.0 ± 7.87 mm in the manual closure group on the ipsilateral side of the tumour subsite (*p* = 0.16) and 18.61 ± 7.52 mm versus 16.64 ± 9.94 mm, respectively, on the contralateral lateral margin (*p* = 0.45). We also noted that neither thyroid cartilage (67 per cent) nor cricoid cartilage (22 per cent) involvement precluded performing a stapler closure. However, stapler closure was avoided in cases of pre-epiglottic space involvement. The other reason for avoiding a stapler is the obvious involvement of the hypopharynx and extension of the tumour to the transitional zone of the larynx. Although we preferred to consider stapler closure in a salvage setting, only 22 per cent had prior irradiation history compared to 45 per cent in the manual closure group. This might have been due to the extra caution taken in this subgroup.

Although the present study was not able to find significant advantages for stapler closure over manual closure for oncological safety, we have noted that stapler closure is not inferior to manual closure in terms of margin safety, two-year mean survival (Figure 2), and surgical costs incurred. A long-term follow up can give further insights into this aspect. There are a few specific considerations while performing stapler-assisted closure of the neopharynx: (1) cricopharyngeal myotomy should be performed, which can be made easier by stretching the pharynx over the indwelling nasogastric tube; (2) tracheoesophageal puncture and primary voice prosthesis insertion can be performed in primary setting using puncture set; and (3) inferior constrictor muscles are sutured over the neopharynx closure site, as the second layer.

- Stapler-closure of laryngectomy defects is oncologically safe for endolaryngeal tumours
- The technique would reduce operating time, minimise post-operative morbidity and help in early recovery
- The technique is avoided when pre-epiglottic space is involved, while the involvement of paraglottic space, cricoid, or thyroid cartilage is not a contraindication for this technique
- The post-operative surgical margin on histopathology specimens in stapler closure was statistically comparable to the manual closure technique, and there was no difference in survival between the two groups
- Primary tracheoesophageal puncture with voice prosthesis insertion is possible in stapler-assisted pharyngeal closure

The present study has its own limitations. The study is of a retrospective nature and more strict and phased time measurements may derive significant operation-time differences between the two types of closure. Objective criteria for diagnosis of pharyngo-cutaneous salivary fistula were not available, hence some cases with minimal pharyngeal breach otherwise undiagnosed may have been missed, leading to underestimation of pharyngo-cutaneous salivary fistula rates. Since stapler closure was adopted recently in our unit, the follow-up period is of lesser duration. Larger prospective and multicentre trials will be able to provide meaningful oncological outcome data for the safety of stapler closure post-laryngectomy.

## Conclusion

Stapler-assisted neopharyngeal closure during laryngectomy appears to be oncologically safe and comparable to manual suturing technique. Careful selection of patients with endolaryngeal disease with no extension to hypopharynx or pre-epiglottic space is mandatory for a good outcome following stapler-assisted closure. There appears to be a reduction in duration of the surgery enabling more efficient utilisation of the operation theatre. Pharyngo-cutaneous salivary fistula rates in the stapler closure group appear to be lower.
